# Editorial: Ticks and Host Immunity – New Strategies for Controlling Ticks and Tick-Borne Pathogens

**DOI:** 10.3389/fimmu.2021.796558

**Published:** 2021-11-18

**Authors:** Ala E. Tabor, Isabel K. F. de Miranda Santos, Nathalie Boulanger

**Affiliations:** ^1^ The University of Queensland, Queensland Alliance for Agriculture & Food Innovation, Centre for Animal Science, St. Lucia, QLD, Australia; ^2^ The University of Queensland, School of Chemistry and Molecular Biosciences, St. Lucia, QLD, Australia; ^3^ Department of Biochemistry and Immunology, Ribeirão Preto School of Medicine, Ribeirão Preto, Brazil; ^4^ UR7290: virulence bactérienne précoce:groupe Borrelia, FMTS, University of Strasbourg, Strasbourg, France; ^5^ French Reference Centre on Lyme Borreliosis, CHRU Strasbourg, Strasbourg, France

**Keywords:** ticks, tick-borne diseases, immunity, vaccines, host genetic resistance

Ticks, as major vectors of human and veterinary diseases, interact with pathogens on two levels: (1) within the tick, where there is a close interaction between the pathogens and the tick’s innate immune system, and (2) during transmission when the pathogens take advantage of the tick’s saliva to increase virulence. The blood feeding habit of ticks also directly affects the host because the parasitic process results in numerous wounds to the host skin and to complete a blood meal ticks inject relatively large amounts of saliva that contain many pharmacological mediators and, depending on the species of the parasite, even lethal toxins. This Research Topic focuses on the host-tick-pathogen interface, adaptations to different hosts, and how these insights can inform the development of successful vaccines and other sustainable technologies for controlling ticks. The 13 articles of this themed Research Topic highlight the latest discoveries and opinions to strategically address tick and tick-borne disease (TBD) control in the future. **Figure 1** is a representation of the articles described.

**Figure 1:**
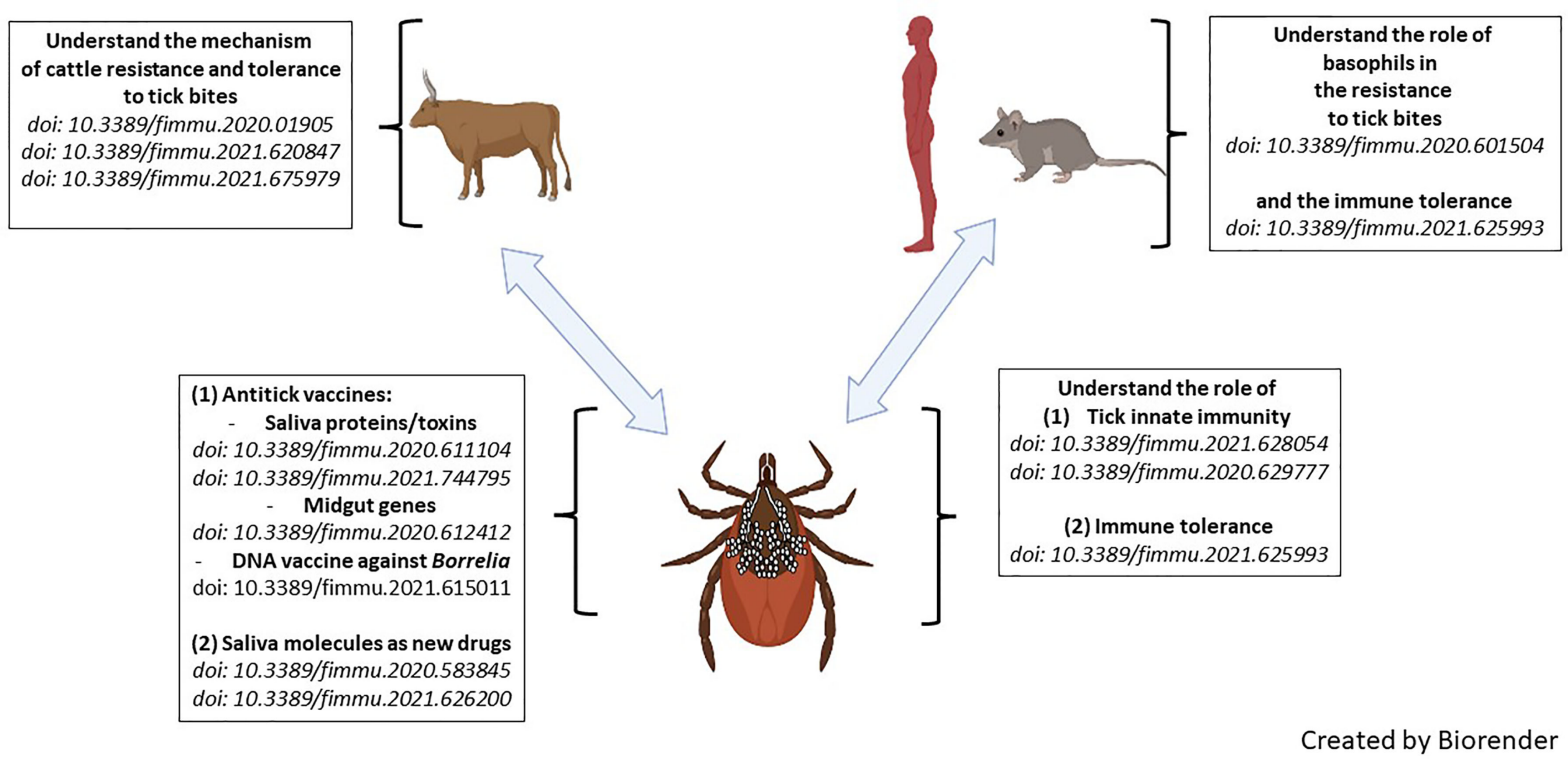
Graphical abstract of articles published in this Research Topic entitled ‘Ticks and Host Immunity – New Strategies for Controlling Ticks and Tick-Borne Pathogens’.

Tick loads in cattle have moderate to high levels of heritability, depending on the population studied and the method used to obtain phenotypes. The advent of technologies supporting genome wide association studies has made the genomic selection of cattle for tick resistance a realistic solution. However, Cardoso et al. point out that genomic selection in cattle for tick resistance has been hampered by the difficulty in phenotyping large numbers of animals for the tick loads necessary to obtain the markers needed for this definitive approach. As an alternative, they pooled genotype and phenotype data from reference populations of beef cattle breeds from three countries that were phenotyped with different methods for tick resistance. For most reference populations this approach is feasible for obtaining genomic estimated breeding values, but accurate phenotypes are fundamental to this approach.

In another study with a genetic approach, Jonsson et al. examine if tick loads in cattle were associated with allelic variants for the tyrosine phosphatase receptor type-C (PTPRC) gene (CD45), a cell surface glycoprotein that initiates antigen receptor signaling in lymphocytes. They did not find a significant correlation with this parameter but did find significant associations between variants and total leucocyte counts, red blood cell counts, and IgG antibody responses to extracts of tick tissues. It will be interesting to examine if these parameters affect parasitism downstream from larval attachment and blood feeding, since leukocytes and hemoglobin contain components that are toxic for the tick and may overload the homeostatic mechanisms that deal with them, reflected by damaged females that oviposit smaller egg masses with lower hatching rates.

Ticks are vectors of economically important hemoparasites for cattle. Cavani et al. examine the relation between heritabilities of tick counts and levels of parasitemia caused by one of these hemoparasites, *Babesia bovis*. As the authors argue, heritability estimates for traits related to infectious diseases are low because the manifestation of the phenotypes relies on many gene products. Indeed, the authors found low heritability (0.077) for parasitemia with *B. bovis*. Nonetheless, among the 42 candidate genes identified with the top 10 SNPs, nine participate in pathways of innate and acquired immune responses. Functional confirmation of their roles in resistance and susceptibility to bovine babesiosis is much anticipated, further to research on genomic selection, they can guide the design of other control measures.

A few articles in this Research Topic explore the identification of novel vaccine targets and modifications in the delivery of vaccines. Klouwens et al. compare the results obtained with recombinant outer-surface protein C (OspC) *Borrelia* vaccination using Freund’s adjuvants (Complete Freund’s for the first boost and Incomplete Freund’s for the second and third boosts) and DNA OspC delivered using a tattoo needle gun. The groups were challenged with *Borrelia burgdorferi* ss (causative agent in human Lyme disease in North America) infected *Ixodes scapularis* nymphs and both were protected from *Borrelia* (except for one positive *Borrelia* culture in the vaccinated group) compared to unvaccinated mice (all positive for *Borrelia*). In contrast, DNA vaccinations for *I. scapularis* genes Salp15, tHRF, TSLPI, and Tix-5 in mice failed to protect against *Borrelia* challenge, despite prior demonstrations of efficacy against tick feeding and *Borrelia* transmission as recombinant vaccines.

*Amblyomma sculptum* is the main tick associated with human bites in Brazil and the vector of Brazilian spotted fever (*Rickettsia rickettsia*). Costa et al. identified three vaccine antigens involved with hematophagy including a Kunitz domain protein, a ‘basic tail’ protein (PFAM domain TSGP1), and an 8.9 kDa polypeptide protein (Von-Willebrand factor type c domain) previously shown to be common in tick saliva. All three proteins were found to be abundant within *A. sculptum* salivary transcriptomes. Recombinant proteins were found to inhibit the activities of factor Xa, thrombin, and/or trypsin. A mouse immunization trial demonstrated 59.4-85% efficacy against adult female ticks and 70-100% against nymphs.

Rodriguez-Valle et al. described the first anti-venom vaccine that successfully protected immunized dogs from paralysis caused by tick holocyclotoxins (HTs). The family of tick holocyclotoxins was recently described in greater detail thanks to functional genomics of tissues from the Australian paralysis tick, *Ixodes holocyclus*. This milestone, together with commercial anti-paralysis sera, permitted the design of synthetic peptides for immunization studies, including evaluating the need for correct folding, a feature that is common to many anti-toxin vaccines, as found also for HTs. With an octavalent vaccine, the authors also addressed the large HT family to ensure coverage of the sequence variability it presents.

A borreliosis study examined the mid-gut transcriptome of *Ixodes ricinus* ticks during the transmission of *Borrelia afzelii* -the predominant Lyme disease agent in Europe. Mahmood et al. identified 553 upregulated and 530 downregulated tick genes in unfed, 24h fed, and fully fed nymphs, with 5 validated in RNA interference experiments. An uncharacterized protein delayed the infection progress and decreased infection prevalence in mouse tissues. The identification of tick proteins associated with *Borrelia* transmission or establishment could help to develop novel preventative strategies for Lyme disease.

Tick saliva is also a key element in tick-borne diseases. Due to its impact on the homeostasis and immunology of the vertebrate host, it contributes to the success of the blood meal but also the transmission of pathogens. Many proteins, peptides, lipids, and non-coding RNA molecules have been identified in saliva. Aounallah et al. review the literature and analyze the immune (i.e. complement system, antibody secretion) and physiological (i.e. itching, pain) mechanisms that are controlled by the saliva. Long non-coding RNAs and miRNAs deserve to be investigated more precisely, especially during secretion into the skin *via* exosomes, given their possible involvement in the regulation of host genes. The authors discuss their potential use in different pathologies and applications in therapeutics. Some saliva proteins are particularly well characterized such as Iripin-3, a serine protease belonging to the serpin superfamily (Chlastáková et al.). It acts at different levels on host adaptive immunity but is also involved with coagulation and macrophage proliferation. (Aounallah et al.).

An extensive review examines the successful synergy between ticks and tick-borne pathogens that leads to host immune tolerance. This facilitates successful tick attachment and feeding, which in turn modulates cutaneous and systemic immune defenses, thus allowing the introduction of the pathogen and contributing to successful long-term infection (Boulanger and Wikel). Gaps identified include the current lack of understanding of skin immunity (including microbiome and non-coding RNAs) to tick-borne pathogens to better unravel the complexity of host-pathogen-tick interactions. Once achieved, we could advance the development of strategies to successfully disrupt both tick feeding and pathogen transmission. Other articles in this Research Topic partly examine these gaps (Mahmood et al.). The resistance of the vertebrate host to ticks has also been studied for many years as a potential way to control ticks, notably the role of basophils in the process of the tick bite (Karasuyama et al.). During this process, the tick saliva proteins are taken up by dendritic cells that migrate to the lymph nodes and induce the activation of B and CD4 memory T cells. IgE antibodies are produced that bind to basophils. During a second infestation, memory CD4 T cells present in the skin release IL-3 that activates basophils and the secretion of histamine. The molecule binds to keratinocyte receptors, which proliferate and form a hyperplasia inhibiting the tick bite.

Many aspects of tick innate immunity have been extrapolated from Drosophila innate immunity studies. Fogaça et al. compare innate immunity mechanisms in insects and ticks highlighting disparities between the two models. Humoral immunity relies upon the activation of antimicrobial peptides, redox metabolism, and the complement system, while cellular immunity is mainly composed of hemocytes involved in encapsulation and nodulation. However, other components of immunity that determine vector competence have been less explored. Rosche et al. reviewed the literature concerning two mechanisms of regulation of homeostasis, notably the “Unfolded Protein Response” and the “Integrated Stress Response”. These processes are well described in mammals and also in insects when responding to viral infections. As ticks are vectors of numerous viruses, it is also reasonable to question the presence of these processes in ticks.

In summary, although major progress has been made in the knowledge of host resistance mechanisms to tick bites, the innate immunity of ticks to pathogens, and the identification of tick saliva molecules essential in the transmission of infectious agents, in large part due to the use of “omics” technologies, many questions remain unanswered and require a multidisciplinary approach to better control ticks and TBDs.

## Author Contributions

All three authors contributed equally to this editorial and approved the submitted version.

## Funding

NB’s research was funded by the French Ministry of Research, Agence Nationale de la Recherche N° ANR-16-CE17-0003-01.

## Conflict of Interest

The authors declare that the research was conducted in the absence of any commercial or financial relationships that could be construed as a potential conflict of interest.

## Publisher’s Note

All claims expressed in this article are solely those of the authors and do not necessarily represent those of their affiliated organizations, or those of the publisher, the editors and the reviewers. Any product that may be evaluated in this article, or claim that may be made by its manufacturer, is not guaranteed or endorsed by the publisher.

